# Psychosocial and occupational factors associated with low back pain among nurses in Saudi Arabia

**DOI:** 10.1002/1348-9585.12126

**Published:** 2020-05-16

**Authors:** Hoda Jradi, Hajjah Alanazi, Yousef Mohammad

**Affiliations:** ^1^ Community and Environmental Health College of Public Health and Health Informatics King Saud Bin Abdulaziz University for Health Sciences King Abdullah International Medical Research Center National Guard Health Affairs Riyadh Saudi Arabia; ^2^ College of Medicine Department of Neurology King Saud University Riyadh Saudi Arabia

**Keywords:** health‐care workforce, LBP, low back pain, nurse, psychosocial factors, Saudi Arabia

## Abstract

**Introduction:**

Low back pain (LBP) is a major health problem with significant public health and economic burden. Few studies have clarified the role of psychosocial factors in LBP occurrence. In this study, we assessed psychosocial and occupational factors associated with LBP, within the last 12 months, among nurses in Saudi Arabia.

**Methods:**

A cross‐sectional study was conducted in 16 hospitals across Riyadh, Saudi Arabia. A sample of 427 nurses was surveyed. The anonymous questionnaire contained valid and reliable questions assessing LBP, as pain between the costal margins of the 12th rib and the gluteal folds, and questions related to psychosocial and work‐related factors experienced by the nurses. Descriptive statistics were reported for all variables. Univariate and multivariate logistic regression analyses assessed the likelihood of significant associations between study variables and LBP.

**Results:**

The prevalence of reported LBP was 80%. Factors associated with LBP in univariate analysis were frequent lifting (OR = 2.32; 95%CI: 1.34‐4.01), work‐related stress (OR = 5.81; 95%CI: 3,37‐9,62), lack of job satisfaction (OR = 2.08; 95%CI: 1.13‐3.85), work‐related problems (OR = 2.40; 95%CI: 1.44‐4.02), and financial problems (OR = 2.08; 95%CI: 1.26‐3.38), while factors that remained significantly associated with LBP in the final multivariate analyses were frequent lifting (OR = 2.04; 95%CI:1.09‐3.81), work‐related stress (OR = 4.22; 95%CI: 2.34‐7.48), and lack of job satisfaction (OR = 1.87; 95%CI: 1.24‐3.58).

**Conclusion:**

The prevalence of LBP is high in this sector of the health‐care workforce. Ergonomic and psychosocial factors may be considered contributing factors for low back pain. Special attention to stress‐reduction, counseling, and policies to improve job satisfaction are recommended in order reduce LBP and improve the health and safety of nurses in Saudi Arabia.

## 1 INTRODUCTION

Lower back pain (LBP) is a major public health problem. Between 70% and 85% of the adult population have reported at least one episode of LBP in their lifetime.^1^ An episode of LBP, defined as the occurrence of pain or discomfort in the spinal area between the costal margins of the 12th rib and the gluteal folds, can lead to persistent medical and non‐medical problems, including disability, loss of productivity, absenteeism, and job changes.^2^ According to the Global Burden of Disease Study (GBD), LBP is considered one of the top 10 conditions contributing to disease and disability, with an estimated number of disability adjusted life years higher than that of human immunodeficiency virus, road traffic injuries, tuberculosis, lung cancer, chronic obstructive pulmonary disease, and pre‐term birth complications.^3^

Nurses are at a higher risk of injuries and work‐related musculoskeletal disorders, such as LBP, than other health‐care professionals.^2^, ^4^, ^5^ Previous studies have shown that there are many risk factors associated with LBP, including demographic, behavioral, and workplace/employment factors.^6^ The demographic and behavioral factors associated with LBP include age, smoking status, and exercise habits.^6^ Other risk factors include seniority in the establishment, professional category, amount of direct patient contact, work posture, necessity of lifting objects or patients, previous LBP training, self‐reported knowledge of LBP, job satisfaction, and job stress.^6^ The psychologic variables associated with LBP include stress, distress, mood and emotions, cognitive functioning, pain behavior, and depressive disorders.^6^

LBP is the leading cause of occupational absence among nurses^7^, ^8^ and the most important reason cited for changing jobs by health‐care workers.^9^, ^10^ In addition, it has been shown that 11% of nurses quit their jobs as a result of LBP.^11^

The reported prevalence of LBP among nurses varies worldwide from 85.7% in England,^11^ to 62% in Italy,^12^ and 80.9% in Hong Kong.^8^ From Africa, a study reported a prevalence of LBP among nurses of 70%.^13^ In 2015, a study conducted in neighboring Qatar, showed a prevalence of LBP among nurses of 54.3%.^14^

Within the Kingdom of Saudi Arabia, as in many other nations, LBP presents a significant issue among healthcare workers, most notably among nurses. The prevalence and risk factors of LBP in Saudi Arabia have been previously investigated in cross‐sectional studies and varied from 48.41% in the Taif region,^15^ to 61% in the Sudayr region,^16^ and 75% in the city of Riyadh.^17^

The epidemiological evidence suggests that the prevalence of LBP is increasing among health professionals and particularly among nurses, making LBP one of the leading causes of disease and disability among this section of the population with a myriad of reported causes depending on the characteristics of study participants and methodology used for assessment of LBP. There is a scarcity of research discussing psychosocial factors such as stress, personal experiences, and life events as they relate to occupational health in the international literature and none in Saudi Arabia. No studies have assessed LBP, as an occupational health issue, from a psychosocial perspective among this growing and essential workforce of nurses in the capital city of Riyadh. Low back pain may have a direct impact on the quality of life of the nurses who in their turn are expected to provide quality healthcare services to their patients. Psychosocial factors may have direct implications for policy making and intervention development to minimize the burden of this serious health problem of major public health impact. This study assessed occupational and psychosocial factors associated with LBP among nurses as a step to take preventive action to alter these modifiable risk factors for better health as a fundamental right for all.

## 2 METHODS

A cross‐sectional study was conducted in 16 hospitals throughout Riyadh. Convenience sampling was applied in this study. After obtaining permission to collect data from the nursing administration at each hospital, a nurse was recruited for every shift to collect data from other nurses working the same shift. The recruited nurse attempted to approach all nurses and ask them to participate in the study after explaining its aims. The same process was applied in all 16 hospitals for duration of 3 months.

## 3 STUDY INSTRUMENT

The instrument used in this study was assembled from valid and reliable tools described in studies published in the English language.^18^, ^19^ The instrument consisted of 63 items. The first section contained general questions about the demographic characteristics of the nurses (age, gender, height and weight, marital status, smoking status, level of physical activity, having children, morbidities, area of practice, level of education, and years of experience). The second section of the instrument examined the prevalence of LBP (ever experiencing an ache or pain in the area of the back situated between the lower margins of the 12th rib and the gluteal folds within the last 12 months), and included questions on treatment (ever receiving treatment for LBP within the last 12 months), its perceived impact on nurses’ work and personal lives, and whether LBP was perceived to be job‐related. The third section contained questions related to working conditions, such as the hours per shift, frequency of shifts, frequency of patient or object lifting, and number of hours spent standing and walking. Other questions were related to perceived stress at work, job satisfaction, plans to change jobs because of LBP, and negative life events during the past year (divorce, death or illness in the family, financial problems, and any other personal problems. The instrument underwent face validity by presentation to a group of five research personnel with nursing backgrounds from different research facilities. The instrument was also tested for clarity, comprehension, and feasibility in a group of 15 nurses that were not included in the study. All suggestions for modifications were taken into consideration. The reliability of the instrument was tested among a group of 30 nurses who answered the questions twice within an interval of 1 week. The test‐retest reliability showed an agreement of 95%. Five hundred surveys were distributed, to which 427 nurses responded. Of the 427 participants, 17 were excluded as a result of incomplete entries, pregnancy, or back surgery. The final response rate was 82.4%.

### 3.1 Data analysis

Analysis of the data was conducted using STATA version 13 (StataCorp LLC, College Station, TX, USA). Categorical data were presented as frequencies. The predicted probability of LBP was calculated using a binary logistic regression model. Univariate and multivariate logistic regressions were conducted to ascertain the effects of study variables reflecting the nurses’ work‐related physical and psychosocial attributes on the likelihood of having LBP. All study variables that showed to be significantly associated with LBP in univariate logistic regression analysis were simultaneously entered in the final model and stepwise backward elimination was applied for all non‐significant variables. The significance level was set at *P* < .05 for all analyses.

## 4 RESULTS

Most of the nurses who participated in this study were women (89.5%) from medical wards. Less than half (42.2%) of them were between the ages of 20 and 30 years, and about 18% were over the age of 40 years. About 29.3% of the participating nurses were overweight and 34.9% of them reported that they did not exercise at all. Two thirds (66.3%) of the nurses had a baccalaureate degree or higher and 43.4% stated that they had more than 8 years of experience. Most (79.5%) of the participants in this study stated that they had LBP, but only 31.9% had been diagnosed by a health‐care professional. Among them, 97.9% said that their LBP is related to their work and 37.1% claimed that they had received treatment for LBP. Results for this section and additional characteristics of the participating nurses are displayed in Table1.

**Table 1:** Characteristics of the study participants (N = 410)

Variable	N (%)
Age group (years)	
20‐30	173 (42.2)
31‐40	163 (39.8)
>40	74 (18.1)
Gender	
Male	43 (10.5)
Female	367 (89.5)
Body mass index (kg/m^2^)	
Underweight (<18.5)	21 (5.1)
Normal weight (18.5‐24.5)	207 (50.5)
Overweight (25‐29.9)	120 (29.3)
Obese (30‐34.9)	62 (15.1)
Educational level	
Diploma	136 (33.2)
Baccalaureate or higher	274 (66.3)
Marital status	
Single	172 (41.9)
Married	225 (54.9)
Separated/Divorced/widowed	13 (3.2)
Experience in nursing (years)	
<2	87 (21.2)
2‐4.9	63 (15.4)
5‐8	82 (20.0)
>8	178 (43.4)
Having children	
Yes	208 (50.7)
No	202 (49.3)
Exercise per week (≥30 minutes)	
None	143 (34.9)
One to two times	210 (51.2)
Three times or more	57 (13.9)
Morbidity	
Yes	42 (10.2)
No	368 (89.8)
Smoker	
Yes	32 (7.8)
No	378 (92.2)
LBP*	
Yes	326 (79.5)
No	84 (20.5)
LBP* is work‐related (N = 326)	
Yes	319 (97.9)
No	7 (2.1)
Treatment for LBP* (N = 326)	
Yes	121 (37.1)
No	205 (62.9)

* LBP, Low back pain.

The majority (43.7%) of the participating nurses reported that they stood/walked for more than 8 hours during their shifts. Reportedly, 94.9% performed lifting of patients and objects; 40.5% said that they performed lifting five times or more during one shift. Regarding night shifts, 76.1% stated that they did seven or more per month. Approximately 62.2% had worked for more than 10 consecutive hours, in their preceding shift.

Almost two thirds of the nurses (63.41%) reported that they were suffering from work‐related stress, 80.24% said that they were unsatisfied with their work, and 43.6% said that they were considering changing jobs because of work problems.

Also, among the surveyed nurses, 11.74% had undergone marital separation in the preceding year and several reported experiencing a death and an illness in the family; 31.1% and 49.0% respectively. Financial problems were reported by 53.9% of the sample. Work‐related activities, psychosocial characteristics, and reported adverse life events of the nurses within the preceding year are displayed in Table 2.

**Table 2:** Work‐related activities, psychosocial characteristics, and reported adverse life events of the participants (N = 410)

Work related activities	N (%)
Standing hours/walking around	
<5	60 (14.6)
5‐8	171 (41.7)
>8	179 (43.7)
Frequent lifting of patients/objects per shift (N = 388)	
<5 times	244 (59.5)
≥5 times	166 (40.5)
Ever training on lifting and transferring patients	
Yes	371 (90.5)
No	39 (9.5)
Night shift frequency/month	
<7 shifts	98 (23.9)
≥7 shifts	312 (76.1)
Working hours/shift	
˂10 hours	155 (37.8)
≥10 hours	255 (62.2)
**Adverse life events**	
Separation/divorce last year	
Yes	48 (11.7)
No	362 (88.3)
Death in the family last year	
Yes	127 (31.0)
No	283 (69.0)
Work‐related problems last year	
Yes	195 (47.6)
No	215 (52.4)
Illness in the family last year	
Yes	200 (49)
No	210 (51)
Financial problems last year	
Yes	221 (53.9)
No	189 (46.1)
Other personal problems last year	
Yes	23 (5.6)
No	387 (94.4)
Took sick leave because of LBP*	
Yes	55 (13.4)
No	355 (86.6)
Considering changing job because of LBP*	
Yes	179 (43.7)
No	231 (56.3)
Job satisfaction	
Yes	81 (19.8)
No	329 (80.2)
Stress at work	
Yes	260 (63.4)
No	150 (36.6)

* LBP, Low back pain.

Results of univariate analysis (Table 3) for factors associated with LBP showed that frequent lifting of patients and objects (≥5 times per shift) (OR = 2.32; 95%CI: 1.34‐4.01), work‐related stress (OR = 5.81; 95%CI: 3.37‐9.62), lack of job satisfaction (OR = 2.08; 95%CI: 1.13‐3.85), work‐related problems in the preceding year (OR = 2.40; 95%CI: 1.44‐4.02), and financial problems in the preceding year (OR = 2.08; 95%CI: 1.26‐3.38) had significant associations with LBP and were simultaneously entered into the multivariate model.

**Table 3:** Results of univariate analysis of factors significantly associated with low back pain

Variable	Reference	P‐value	OR	Lower	Upper
Work‐related activities					
Frequent lifting of objects and patients	No frequent lifting	0.003	2.32	1.34	4.01
Psychosocial factors					
Work‐related stress	No stress	0.001	5.81	3.37	9.62
Lack of Job satisfaction	satisfaction	0.018	2.083	1.13	3.85
Work problems (past year)	No problems	0.001	2.4	1.44	4.02
Financial problems (past year)	No problems	0.004	2.083	1.26	3.38

Abbreviations: BMI, body mass index; OR, odds ratio.

Table 4 shows the final results of the multivariate analysis for factors that remained significantly and independently associated with LBP. Frequent lifting of patients and objects (≥5 times/shift) (OR = 2.04; 95%CI:1.09‐3.81), work‐related stress (OR = 4.22; 95%CI: 2.34‐7.48), and lack of job satisfaction (OR = 1.87; 95%CI: 1.24‐3.58) were independently and significantly associated with LBP. The final model was shown to be a good fit by the Hosmer–Lemeshow test (*P* > .05).

**Table 4:** Results of the multivariate analysis for factors associated with low back pain

Variable	Reference	P‐value	OR	Lower	Upper
Work‐related stress	No stress	<0.001	4.22	2.34	7.48
Lack of job satisfaction	Satisfaction	0.041	1.87	1.24	3.58
Frequent lifting objects and patients	No frequent lifting	0.026	2.04	1.09	3.81

Abbreviations: CI, confidence interval; OR, odds ratio.

## 5 DISCUSSION

This study demonstrated that along with the ergonomic factor of frequent lifting, work‐related stress, lack of job satisfaction, work‐related problems, and financial problems were associated with LBP among this group of nurses. The role of frequent lifting and lack of job satisfaction in multivariate logistic regression remained significantly high; however work‐related stress showed the most prominent association with LBP.

Previous studies reported on work conditions and nurses’ personal characteristics as factors associated with LBP. In this study, we assessed personal and behavioral characteristics such as age, level of education, smoking status, obesity, and job‐related factors such as years of experience, work schedule, and work‐related activities. We also focused on psychosocial factors such as hazards for LBP. Considering work‐related stress and lack of job satisfaction as psychosocial risk factors, it is expected that by implementing appropriate interventions to reduce stress in the workplace and increasing job satisfaction, the occurrence of LBP will be reduced.

In this current study the prevalence of LBP is 80%. Regardless of the criteria used to assess LBP in each study, this prevalence is almost similar to reported prevalence from Taiwan (82%)^20^ and Malaysia (79.4%),^21^ and higher than reported prevalence from neighboring Yemen (61%),^22^ Greece (75%),^23^ Nigeria (73.5%),^13^ and the Netherlands (62%).^24^ In Saudi Arabia, a previous study among nurses from the Sudayr region and another among healthcare from the Taif region reported prevalence of LBP of 61%^16^ and 48.14%^15^ respectively. Both studies assessed low back pain by simply inquiring if the nurses suffered from pain in the lower back with no specifics regarding the location of the pain. The differences in the prevalence of LBP between these studies may reflect variations in the assessment method of LBP, can be attributed to differences in reporting, or to differences in cultural perception and tolerance of pain.

Most nurses in this study performed frequent lifting of objects and patients. In accordance with previous studies, conducted among nurses, reported frequency of lifting showed to be significantly associated with LBP.^21^, ^25^ Additionally, a study by Barrero et al^26^ and a study by Smedley et al^24^ reported that lifting was an important exposure variable associated with LBP. High workloads and poor working conditions may require nurses to perform tasks, such as heavy lifting, that will place them at risk for developing LBP.^27^ It is worth mentioning that most of the nurses surveyed in this study were women (89.49%), and this may reflect a shortage of male nurses in the health‐care system in which the study was conducted. Female nurses are most likely to be pressured to perform strenuous activities and physical work that may explain the high prevalence of LBP in this group.

Lack of job satisfaction and work‐related stress were the two psychosocial factors significantly associated with LBP in this group of nurses. Consistent with our results, low job satisfaction has been significantly associated with LBP among nurses.^19^, ^21^ Across the literature, epidemiologic evidence has shown that LBP is associated with physical as well as psychologic risk factors.^23^, ^24^ The reasons underlying low job satisfaction, as a psychosocial risk factor, in developing countries have included lack of job security, poor salaries, fewer benefits, poor working conditions, lack of promotion, poor employee–administration communication, lack of control over the job, poor leadership behaviors, job stress, and poor mental and physical status.^24^, ^28^

Further research is recommended regarding all the factors that were attributed to low job satisfaction and how do they apply to the nursing profession.

Work‐related stress was significantly associated with LBP in this study. In previous studies, nurses who reported LBP and high level of stress, also reported reduced productivity and more absenteeism.^21^ The phenomenon of the stress in the workplace, its mediating factors, and how it relates to nurses’ health still must be explored. Nurses may continue to work regardless of their lack of job satisfaction and stressful work conditions out of fear of losing their source of income. Lack of job security and poor salaries have been cited to be associated with ill health.^29^ Financial problems and work‐related showed to be significantly associated with LBP among this sample of nurses in univariate logistic regression, further suggesting a pathway linking job satisfaction, financial hardships, and LBP.

This study has some limitations that should be acknowledged. First, there is a lack of standardized measures for LBP among studies to be able to make a solid comparison with previous findings related to this topic. Second, with this cross‐sectional study design, the exposure and outcome were assessed simultaneously and no evidence of a temporal relationship between exposure (such as work‐related stress and lack of job satisfaction) and outcome (LBP) can be established. Moreover, the data were collected using a self‐reported questionnaire, which is inherently biased by the person's feelings at the time they filled it out.

## 6 CONCLUSION

The prevalence of LBP is higher than ever reported in this important sector of the health‐care workforce. Stress reduction strategies, stress counseling, and policies related to improving job satisfaction, as evidence‐based interventions for LBP among nurses, should be implemented to reduce the prevalence of LBP in Saudi‐Arabian nurses. Furthermore, frequent ergonomic training on how to work safely should be introduced to improve the health and safety of nurses.

## DISCLOSURE

*Ethical approval:* The research was approved by the local ethics committee at King Saud Bin Abdulaziz University for Health Sciences. All procedures performed in this study were in accordance with the ethical standards of the institutional and/or national research committee and with the 1964 Helsinki declaration and its later amendments or comparable ethical standards. *Informed consent:* Informed written consent was obtained from all individual participants included in the study. *Registry and the registration number of the study:* King Abdullah International Medical Research Center (KAIMRC) number SP14/084). *Animal studies:* Not applicable. *Conflict of interest:* The authors declare that they have no conflict of interest.
